# Giant Cell Myocarditis: Is It Time to Redeploy Cytokine Inhibitors?

**DOI:** 10.31138/mjr.240725.eca

**Published:** 2026-03-01

**Authors:** Deepak Nagra, Iyantha Sarindhi Kuruppurachchi, Kaiyang Song, Mark Russell, Katie Bechman, Maryam Adas, Zijing Yang, Chamith Rosa, Siwalik Banerjee, Olivia Flowers, Chris Wincup, Arti Mahto, Daniel Bromage, Nicola Gullick, James Galloway

**Affiliations:** 1 Centre for Rheumatic Disease, King’s College London - Centre for Rheumatic Disease, United Kingdom;; 2 Department of Rheumatology, University Hospitals Coventry & Warwickshire, United Kingdom

**Keywords:** giant cell myocarditis, autoimmune, inflammation, JAK inhibition, cytokine inhibitors, monoclonal antibody

## Abstract

Giant cell myocarditis (GCM) is a rare, aggressive T-lymphocyte-mediated inflammatory cardiac disorder associated with high mortality. Diagnosis relies on endomyocardial biopsy, revealing lymphocytic infiltrates, granulomas, and myocyte necrosis, with cardiac MRI serving as a valuable non-invasive adjunct. Due to its rarity and coding limitations, GCM remains poorly captured in epidemiological data, hindering robust clinical trials. Current treatment strategies are largely based on case reports and off-label immunosuppressive use, with limited success using conventional DMARDs. Emerging evidence supports targeting T-cell-mediated and cytokine-driven pathways, including TNF inhibitors, JAK inhibitors (e.g., tofacitinib, ruxolitinib), and IL-2-targeted therapies (e.g., basiliximab). Tofacitinib, in particular, offers rapid, broad-spectrum immunomodulation and favourable pharmacokinetics. Although high-quality evidence is lacking, real-world data and coordinated international efforts (e.g., multicentre case series, registries) may help define optimal therapeutic strategies. With increasing availability of biosimilars and off-patent biologics, early cytokine inhibition could represent a paradigm shift in the management of this life-threatening condition.

With the increasing availability of cardiac imaging modalities, such as cardiac MRI (cMRI), autoimmune and autoinflammatory cardiac disorders have emerged as significant disease entities. Giant cell myocarditis (GCM) is a rapidly progressive, T-lymphocyte-mediated disease that can result in end-stage cardiac failure and death. Common presentations include decompensated heart failure, ventricular arrhythmias, cardiopulmonary arrest, and sudden cardiac death. The gold standard diagnostic test for GCM is endomyocardial biopsy, which typically demonstrates dense lymphocytic infiltrates and immature granulomas positive for CD68 and CD31, alongside myocyte necrosis.^1^ Myocyte necrosis helps to differentiate GCM from cardiac sarcoidosis, which typically lacks necrosis and other extra-cardiac manifestations. Classic cMRI findings in GCM include subendocardial perfusion defects, with delayed gadolinium enhancement.

GCM is exceptionally rare, and there are no available contemporary estimates of its prevalence in the UK. Analysis of hospital admissions for GCM is hindered by the condition being grouped under the broader category of ‘other myocarditis’ in diagnostic coding.^2^ According to publicly available NHS Hospital Episode Statistics, there were 34 admissions recorded under this category in 2023–2024. The scarcity of GCM (reported in <0.007% from a Japanese autopsy registry),^3^ alongside ethical and practical challenges in diagnosis, have precluded the development of treatment strategies that are based on high-quality evidence (e.g. randomised control trials). There is a reliance on off-label immunosuppressive use, case reports,^4^ and anecdotal data for treatment guidance. Conventional DMARDs, such as tacrolimus, mycophenolate, methotrexate and azathioprine, have been widely utilised with limited success,^5^ with one case series demonstrating a transplant-free survival rate of 42% with DMARD-only treatment.^6^ The coupling of tacrolimus with amiodarone also poses the risk of QTc prolongation making this yet another therapeutic challenge.

There is clear evidence that giant cell myocarditis is driven by the interplay between macrophages and T lymphocytes. This is supported not only by histological findings, but also by gene expression profiles implicating T cell activation and associated cytokine signatures^7^ with CD68 macrophages secreting tumour necrosis factor and interlukin-1. Taken together, these findings support the rationale for targeting T cell-mediated (particularly Th1) pathways.^8^ Given the catastrophic nature of the disease, current strategies often rely on potent immunosuppression — yet outcomes remain poor and mortality high. Conducting a conventional clinical trial will likely be unfeasible due to disease rarity. Instead, coordinated international efforts using N-of-1 designs or multicentre case series could allow us to evaluate candidate therapies systematically.

Following their introduction for the treatment of rheumatoid arthritis over two decades ago, the use of tumour necrosis factor inhibitors (TNFi) has expanded rapidly to include seronegative inflammatory arthropathies, inflammatory bowel disease, inflammatory skin disease, and more recently, multisystem sarcoidosis. While TNFi historically carried cautionary labels for use in heart failure, recent studies have demonstrated promising outcomes in cardiac sarcoidosis (CS).^9^ Given some similarities in presentation between GCM and CS, there is increasing interest in evaluating whether TNFi could be a viable treatment option for GCM. To date, a single case report has described the use of a TNFi, etanercept, in GCM,^10^ with a further review suggesting a role for infliximab.^11^ In this report there had been initial attempts for disease induction with conventional disease modifying anti-rheumatic drugs and cyclophosphamide, whilst TNFi was utilised as a fourth-line agent; however, despite this, the patient died whilst he was being prepared for a heart transplant.

There is now a broad array of off-patent and readily available highly targeted T cell inhibitors, across both biologic and small molecule therapies. Case reports have described use of TNFi, IL-2-targeted monoclonals, and JAK inhibitors. JAK inhibition, particularly high-dose tofacitinib, offers a strategically rational first-line approach. The signalling of IL-2 through JAK3-STAT5 is crucial for T Regulatory (Treg) cell stability.^12^ Tofacitinib has been shown to provide an anti-inflammatory impact, without a change in Treg cell abundance.^12^ Tofacitinib has a rapid onset and offset, offering flexibility in cases of toxicity or non-response.^13^ Tofacitinib is effective at 10 mg BID in suppressing canonical interferon pathways central to the pathogenesis of GCM.^14^ When compared to tacrolimus, tofacitinib has demonstrated an added advantage with increased tissue penetration and a broader range of cytokine inhibition.^14^ It is readily accessible and increasingly affordable, with patent expiry due in 2025. Limiting factors for the use of JAK inhibitors include their infection and cardiovascular risk profile with increased rates of shingles and myocardial infarctions noted in a population of rheumatoid arthritis, however this may be less relevant in the context of GCM.

Ruxolitinib, a JAK 1/2 inhibitor, blocks downstream cytokines including IL-2, IL-4, IL-6, and TNF. Its utility in GCM has been explored in case reports,^5^ in which a cardiac transplant was avoided due to control of disease activity. Another potential treatment is basiliximab, a monoclonal antibody targeting the IL-2 receptor, initially used in hepatic and renal transplantation. Originally developed by Novartis, basiliximab is now off-patent, making it a more accessible option with case reports on its use in refractory GCM, particularly in patients requiring cardiac transplantation.^15–18^ In both cases, basiliximab was used following cardiac transplantation as a fourth- or fifth-line agent following conventional disease modifying anti-rheumatic drugs and prednisolone use (**Table 1**).

**Table 1.** Baseline characteristics of those treated with cytokine inhibitors for giant cell myocarditis with outcomes.

**Author (Year)**	**Age (Gender)**	**Presentation**	**Investigation**	**Treatment**	**Outcome**
Nash (2001)^10^	46 (M)	Chest pain and shortness of breath – 5 hours	ECG: anterior ST elevation, ECHO: LVEF 19%, EMB: GCM	Prednisolone IVIG 126mg Cyclophosphamide 375mg/m^2^/day for two days. Etanercept 50mg weekly. Azathioprine 50mg day with re-introduction of Cyclophosphamide.	Death, enroute to transplant.
Anderson (2013)^18^	41 (F)	Chest pain with shortness of breath, in cardiogenic shock on presentation.	ECG: not reported ECHO: LVEF 30%, EMB: GCM	Initially managed for Lymphoma with RCHOP for 14 months prior to presentation. Subsequently received Prednisolone and Cyclosporin for GCM; followed by heart transplant with anti-thymocyte globulin and further Steroids, Mycophenolate Basiliximab and Tacrolimus.	Death 4 weeks later due to multi-organ failure following transplant.
Mitoff (2013)^17^	25 (F)	Shortness of breath and chest pain.	ECG: ST elevation anterior leads ECHO: LVEF 20%	Previous Hydroxychloroquine, Sulfasalazine and Prednisolone for seronegative Rheumatoid Arthritis. Induction with methylprednisolone, IVIG, Basiliximab followed by Mycophenolate, Tacrolimus and oral Prednisolone.	Remission after 7 months.
Bowman (2024)^20^	20 (F)	Chest pain	ECG: ST elevation V1-V2 ECHO: LVEF <20%, EMB: GCM	Methylprednisolone Ruxolitinib followed by Tacrolimus.	Remission.
Xiong (2024)^21^	20 (F)	Chest pain 2 days	ECG: Anterior ST elevation, ECHO: LVEF <20%, EMB: GCM	ECMO & Supportive care, Tacrolimus 3mg BD, Prednisolone 50mg OD, Ruxolitinib 10mg BD	Recovery with discharge.

The high cost of monoclonal antibodies has historically meant that they were recommended as later-line treatments for many diseases in national and international guidelines. However, with the advent of biosimilars and reduced cost, these agents are increasingly used earlier in the treatment paradigm in inflammatory diseases. Mouse models of GCM have demonstrated benefit of cytokine inhibition with interluking-1 blockade.^19^ Given the high mortality rate of GCM, an important question remains: should JAK inhibitors be considered first-line therapies to improve outcomes in individuals with GCM?

In the absence of a randomised controlled trial exploring this important question, real-world data is needed to provide further insight into the role of cytokine inhibitors in GCM management. Studies should focus on consolidating case reports and retrospective data, while prospective studies (e.g. registries for rare disease) will help to better define the efficacy and safety of TNFi and other cytokine-targeting therapies in GCM.

## Data Availability

The data underlying this article are available in the article and in its online supplementary material.
